# Health profiles of foreigners attending primary care clinics in Malaysia

**DOI:** 10.1186/s12913-016-1444-0

**Published:** 2016-06-14

**Authors:** Norazida Ab Rahman, Sheamini Sivasampu, Kamaliah Mohamad Noh, Ee Ming Khoo

**Affiliations:** Healthcare Statistics Unit, Clinical Research Centre, Ministry of Health, Kuala Lumpur, Malaysia; Family Health Development Division, Ministry of Health, Putrajaya, Malaysia; Department of Primary Care Medicine, Faculty of Medicine, University of Malaya, Kuala Lumpur, Malaysia

**Keywords:** Foreigners, Health services, Primary care, Health-profile, Malaysia

## Abstract

**Background:**

The world population has become more globalised with increasing number of people residing in another country for work or other reasons. Little is known about the health profiles of foreign population in Malaysia. The aim of this study was to provide a detailed description of the health problems presented by foreigners attending primary care clinics in Malaysia.

**Methods:**

Data were derived from the 2012 National Medical Care Survey (NMCS), a cross sectional survey of primary care encounters from public and private primary care clinics sampled from five regions in Malaysia. Patients with foreign nationality were identified and analysed for demographic profiles, reasons for encounter (RFEs), diagnosis, and provision of care.

**Results:**

Foreigners accounted for 7.7 % (10,830) of all patient encounters from NMCS. Most encounters were from private clinics (90.2 %). Median age was 28 years (IQR: 24.0, 34.8) and 69.9 % were male. Most visits to the primary care clinics were for symptom-based complaints (69.5 %), followed by procedures (23.0 %) and follow-up visit (7.4 %). The commonest diagnosis in public clinics was antenatal care (21.8 %), followed by high risk pregnancies (7.5 %) and upper respiratory tract infection (URTI) (6.8 %). Private clinics had more cases for general medical examination (13.5 %), URTI (13.1 %) and fever (3.9 %). Medications were prescribed to 76.5 % of these encounters.

**Conclusions:**

More foreigners were seeking primary medical care from private clinics and the encounters were for general medical examinations and acute minor ailments. Those who sought care from public clinics were for obstetric problems and chronic diseases. Medications were prescribed to two-thirds of the encounters while other interventions: laboratory investigations, medical procedures and follow-up appointment had lower rates in private clinics. Foreigners are generally of young working group and are expected to have mandatory medical checks. The preponderance of obstetrics seen in public clinics suggests a need for improved access to maternal care and pregnancy related care. This has implication on policy and health care provision and access for foreigners and future studies are needed to look into strategies to solve these problems.

## Background

The number of people moving from one country to another has increased in recent years, especially in South East Asia; approximately 30 % of the population in Singapore and 8 % in Hong Kong were foreigners in 2014 [1, 2]. In Malaysia, the number of foreigners increased from 1.4 million in 2000 to 2.3 million in 2010; the latter comprised 8.2 % of the 28.6 million population in Malaysia [3]. However, this number was probably an underestimation because there were a number of undocumented foreigners who might not have been included [4]. It was estimated that more than 90.0 % of these foreigners were low to medium-skilled foreign workers [5, 6]. The remaining were expatriates, foreign spouses, international students, foreign retirees who migrated to Malaysia via international residency scheme (Malaysia My Second Home Programme), asylum seekers, and refugees that altogether constituted less than 300 000 people [4–7]. Malaysia also received on average 2 million tourists per month which totalled to 25.03 million tourists in year 2012 [8].

The health of these foreigners has generated much interest because of their diverse problems and health seeking behaviours [9–12]. In general, foreigners are expected to be healthy when they enter another country due to self-selection process and pre-departure health screening; this is commonly described as the “healthy immigrant effect” [13, 14]. However, their health could deteriorate over time, thereby necessitates the use of healthcare services [13, 15, 16]. Previous studies have indicated that 46.0 to 57.0 % of foreign workers experienced health problems while working or during their stay abroad [11, 17, 18]. Being mostly manual workers, they are at risk of occupational accidents and diseases [18, 19]. Furthermore, their poor living conditions and inadequate access to healthcare facilities compound the problem which could therefore pose a public healthcare issue [4, 20, 21].

To our knowledge, no research so far has been undertaken to examine access to health services of foreigners in Malaysia. The dual healthcare system in Malaysia comprising the public and private sector provides comprehensive range of services and access to healthcare. The public healthcare system is heavily subsidised by the government and designed on the basis of providing affordable healthcare. Private healthcare on the other hand, operates on a fee-for-service basis and is limited to those who can afford it as it is relatively more costly compared to public healthcare services. All foreign workers in Malaysia are required to subscribe to basic private health insurance scheme that covers hospitalisation and surgical charges at public hospitals [22, 23]. Besides this compulsory health insurance, some employers also provide additional medical benefits for the workers, although the extent of coverage varies. It is also mandatory for international students to have private health insurance to cover the cost of medical care during their stay in Malaysia [24]. The 2012 statistics reported that 826,801 foreigners attended public healthcare facilities throughout country; the numbers showed an increment of 16.5 % from the previous year [25]. Of these, 420,722 were visit to public primary care clinics and they comprised 1.4 % of total primary care attendances for 2012. Under the medical tourism programme in Malaysia, the number of medical tourists in 2011 was 578,403 and this figure is directed to private hospitals in Malaysia [26]. The number of foreigners who visited private clinics was unknown.

The frequency and range of health problems encountered by foreigners provides a description of healthcare services utilisation and needs. Yet, little is known about the morbidity and utilisation patterns by foreigners in primary care, and no detailed description is available for Malaysia. Such information would provide an insight into their needs and access to healthcare services hitherto not realised. This study aimed to describe foreigners’ utilisation of primary healthcare specifically the characteristics of foreign patients in primary care clinics in Malaysia and the health problems commonly encountered.

## Methods

### Setting

Primary care clinics in Malaysia functions as a partial gatekeeper to secondary care, although people can bypass a referral from public or private practitioners and go directly to specialist and hospital. In 2008, public clinics handled 38 % of total primary care visit despite accounting for only 11 % of primary care clinics in Malaysia [27]. By December 2012, there were 871 public primary care clinics and 5198 private clinics (general practitioners) [28]. The government-run public healthcare facilities are accessible to foreigners, but at a higher cost compared to Malaysian citizens due to lower subsidised rate imposed on foreigners. Up until 2014, foreigners were charged MYR15 (USD3.5) for primary care consultation and MYR60 (USD14) for consultation with specialist while Malaysian citizens pay MYR1 (USD0.2) and MYR5 (USD1.2), respectively [29]. On the other hand, standard consultation fees at private clinics range from MYR20 (USD4.6) to MYR50 (USD11.6) per visit.

### Data source and patient identification

We used data from the National Medical Care Survey (NMCS) that collected information on primary care encounters from public and private primary care clinics in Malaysia. The NMCS was a cross-sectional survey carried out from August to November in 2012 and surveyed primary care clinics from five regions in Malaysia (Kuala Lumpur, Selangor and Putrajaya, Kelantan, Kota Kinabalu, and Kuching) [30]. These regions were selected to represent all parts of Malaysia. From the list of primary care clinics obtained from Ministry of Health Family Health Development Division and the Private Medical Practice Division, the public and private primary care clinics were selected through stratified randomised sampling by sector and regions. Outpatient clinics within hospital, specialist clinics, and clinics without permanent doctors were excluded from the study frame. The sample size was determined by calculating the number of encounters needed based on estimation of prevalence rate of variable of interest, power of 80 %, 95 % confidence interval and adjusted for the design effect and expected response rate from each sector; 70 % for public clinics and 30 % for private clinics [31]. The study sample consisted of 75 public clinics and 383 private clinics.

Each clinic was given a randomly selected date for data collection; weekends and public holidays were excluded. Using a data collection form, doctors in each clinic recorded details of patient encounters during or after the consultation with the patients. These included (i) socio-demographic data (ii) reasons for the encounter (RFEs) i.e., for symptoms, follow-up or for procedural/administrative reasons (iii) diagnosis or clinical problem managed (iv) prescription and/or any other interventions ordered. RFE refers to patient’s complaint or reason for seeing a doctor, while diagnosis was recorded based on diagnosis made by doctors during the encounter. Diagnoses, investigations and procedures were coded according to the International Classification of Primary Care Version 2 (ICPC-2) [32]. Medications were coded according to the Anatomical Therapeutic Chemical (ATC) classification [33]. Data were coded by trained data entry personnel and quality check on data entry was done through double data entry. Patients who consulted doctors for different reasons twice within the same day were counted as having two encounters.

From the NMCS records, we identified all patient encounters who are foreigners. A foreigner was defined as any patient with foreign nationality (non-citizen). We excluded permanent residents because details on nationality for this group of patients were not available.

### Data analysis

The NMCS used a cluster sample design with primary care clinics used as the primary sampling unit and patient encounter as the unit of analysis. The data were weighted to adjust for over or under representativeness of strata and non-response by applying sampling weight and post-stratification weight. Sampling weight was calculated based on the inverse probability of selecting a unit and post-stratification weight as an adjustment for non-response was calculated by dividing total number expected with the total respondents [30]. Analysis was done using the survey function in STATA version 13 Statistical Software (College Station, TX: StataCorp LP) [34, 35]. Results were presented as number of observations and percentages. Chi-square test was applied to compare proportions between sector and p-value of <0.05 was considered as significant.

### Ethical approval

The NMCS was approved by the Medical Research and Ethics Committee of the Ministry of Health Malaysia (NMRR-09-842-4718). A public notice was placed at each participating clinics to inform patients of the ongoing study and that data would be collected for research purposes.

## Results

Sixty-nine public clinics and 120 private clinics participated in NMCS, resulting in a response rate of 92.0 and 31.3 % for public and private sector respectively. A subsequent analysis comparing physicians’ characteristics between respondents and non-respondents for private clinics was performed and no significant differences in terms age, gender and years of practice were observed (Appendix). In all, there were 141,593 encounters for NMCS; 42,340 (29.9 %) were from the public clinics and 99,253 (70.1 %) were from the private clinics. Of these, foreigners constituted 10,830 or 7.7 % of all patient encounters; 1067 or 2.5 % of encounters in public clinics and 9763 or 9.8 % of encounters in private clinics.

Table 1 shows demographic features of the study population. More females were encountered in public clinics (60.2 %) compared to private clinics (26.7 %). When analysis was repeated to exclude gender specific health problems (obstetric, gynaecological, and andrological conditions), females accounted for 35.2 and 24.5 % of encounters in public and private clinics respectively. Patients in private clinics were younger (median age, 28.2) than those in public clinics (median age, 30.0), but the differences were slight. Patients aged 20 to 39 years old accounted for 64.9 % of encounters in public clinics and 79.8 % encounter in private clinics. Altogether 31 different nationalities were identified. The top four were from Indonesia, Bangladesh, Nepal and Myanmar, which constituted 72.4 % of the study sample.

**Table 1:** Demographic characteristics of patient encounters

	Overall		Public clinics		Private clinics		
Characteristic	No. of encounter^a^	Percentage	No. of encounter	Percentage	No. of encounter	Percentage	*P-*value^*^
Gender							
Male	6569	69.9	379	39.7	6190	73.3	<0.001
Female	2831	30.1	574	60.2	2257	26.7	
Age (years)							
Median, IQR	28.3	(24.0, 34.8)	30.0	(23.3, 36.5)	28.2	(24.0, 34.5)	
Age group							
< 1	305	3.1	111	11.3	194	2.2	<0.001
1–9	109	1.1	20	2.0	89	1.0	
10–19	268	2.7	28	2.9	240	2.7	
20–29	4832	48.7	329	33.5	4503	50.4	
30–39	2920	29.4	308	31.4	2612	29.2	
40–49	1182	11.9	125	12.7	1057	11.8	
50–59	209	2.1	34	3.4	175	2.0	
≥ 60	100	1.0	27	2.7	73	0.8	
Country of origin							
Asia	9023	96.1	988	99.4	8035	95.7	0.1603
Indonesia	2355	25.1	301	30.2	2054	24.5	
Bangladesh	1751	18.7	131	13.2	1620	19.3	
Nepal	1505	16.0	85	8.5	1420	16.9	
Myanmar	1182	12.6	90	9.1	1092	13.0	
India	544	5.8	39	4.0	505	6.0	
The Philippines	440	4.7	153	15.4	287	3.4	
Others^b^	1246	13.3	189	19.0	1057	12.6	
Africa	226	2.4	6	0.6	220	2.6	
Europe	102	1.1	-	-	102	1.2	
Oceania	37	0.4	-	-	37	0.4	

Note: *IQR* interquartile range

^*^Chi-square test for differences between public and private clinics, P-value < 0.05 considered as significant

^a^Total encounter may not total up to 10,830 because of missing information

^b^Other Asia countries include Pakistan, Thailand, Vietnam, China, Cambodia, Sri Lanka, Singapore, Iran, Iraq, Laos, Syria, Timor, and Yemen

Table 2 describes the commonest reasons for encounter (RFEs) by three sub-categories: symptoms, follow-up and procedure/administrative. Most encounters were for symptoms of acute illnesses such as fever, cough and abdominal pain. Compared to private clinics, encounters who came for follow-up visit and procedural/administrative purposes were more often in public clinics. Of all RFEs, antenatal check-up was the primary RFEs in public clinics (29.0 %) whereas symptoms of fever (12.5 %) and cough (11.8 %) were the commonest RFEs in private clinics.

**Table 2:** Distribution of common patients’ reasons for encounter

	Overall		Public clinics		Private clinics		
Reasons for encounter (RFEs)	n	Percentage	n	Percentage	n	Percentage	P-value^*^
Symptom	11,297	69.5	606	41.3	10,691	72.3	0.0004
Fever	1941	11.9	97	6.6	1844	12.5	0.0192
Cough	1869	11.5	122	8.3	1747	11.8	0.1563
Abdominal pain	896	5.5	36	2.4	860	5.8	0.0066
Musculoskeletal symptoms	524	3.2	67	4.6	457	3.1	0.3262
Back problems	412	2.5	-	-	412	2.8	-
Follow-up	1209	7.4	300	20.4	909	6.1	0.0010
Hypertension	297	1.8	77	5.3	220	1.5	0.0070
Diabetes mellitus	251	1.5	76	5.2	175	1.2	0.0061
Asthma	128	0.8	6	0.4	122	0.8	0.4919
Lipid disorder	73	0.4	36	2.4	37	0.2	0.0069
Osteoarthritis	65	0.4	-	-	65	0.4	-
Procedure/administrative	3744	23.0	562	38.3	3182	21.5	0.0416
General medical examination	1262	7.8	23	1.6	1239	8.4	0.0020
Diagnostic radiology/imaging	908	5.6	51	3.5	858	5.8	0.3871
Antenatal check-up	670	4.1	426	29.0	244	1.7	<0.0001
Blood test/investigation	311	1.9	21	1.4	290	2.0	0.6979
Dressing	276	1.7	24	1.7	252	1.7	0.9651
Total RFEs	16,250	100	1468	100	14,782	100	

Note: General medical examination - include routine medical check-up, pre-employment & pre-university check-up

*Chi-square test for differences between public and private clinics, P-value < 0.05 considered as significant

The 10,830 encounters resulted in 13,008 diagnoses, with an average of 120 diagnoses per 100 encounters (135 per 100 encounters for public clinics and 118 per 100 encounters for private clinics). The frequency and percentage of the commonest 20 diagnoses in public and private clinics are presented in Table 3. The commonest diagnosis in public clinics was antenatal care, accounting for 21.8 % of all diagnoses. This was followed by high risk pregnancies (7.5 %), upper respiratory tract infection (URTI) (6.8 %), hypertension (6.5 %) and diabetes mellitus (5.3 %). As for private clinics, general medical examination was most common (13.5 %) followed by URTI (13.1 %), fever (3.9 %) trauma/injury and accidents (3.6 %), and fungal infection (3.6 %). Of total diagnoses, pregnancy-related conditions (obstetric cases) constituted 37.7 % of diagnoses in public clinics and 2.4 % of private clinics. Chronic non-communicable diseases, namely hypertension, diabetes mellitus and lipid disorder comprised 15.5 % and 4.5 % of diagnoses in public and private clinics respectively. There were six encounters diagnosed with tuberculosis and all of them presented to the public clinics. In addition, there were 45 encounters of tuberculosis suspected cases; 17 in public clinics and 28 in private clinics.

**Table 3:** Top 20 diagnoses/problems managed

		Public			Private
Diagnosis	n	Percentage	Diagnosis	n	Percentage
Antenatal care	316	21.8	General medical examination	1781	13.5
Pregnancy high risk	108	7.5	Upper respiratory infection	1515	13.1
Upper respiratory infection	98	6.8	Fever	448	3.9
Hypertension	94	6.5	Trauma/injury/accident	420	3.6
Diabetes mellitus	76	5.3	Fungal infection	412	3.6
Gestational diabetes	62	4.3	Musculoskeletal symptoms	412	3.6
Lipid disorder	53	3.7	Gastroenteritis	384	3.3
Neonatal jaundice	42	2.9	Gastritis	375	3.2
Anaemia	42	2.9	Back problems	330	2.9
Toxaemia of pregnancy	31	2.1	Hypertension	248	2.1
Trauma/injury/accident	31	2.1	Cough	232	2.0
Cystitis/urinary infection	25	1.8	Asthma	232	2.0
Weight gain	21	1.5	Headache	220	1.9
Gastritis	17	1.2	Teeth/gum symptoms	220	1.9
To rule out tuberculosis	17	1.2	Dermatitis	192	1.7
Laceration/cut	16	1.1	Conjunctivitis	184	1.6
Gastroenteritis	16	1.1	Diabetes mellitus	175	1.5
General medical examination	14	1.0	Antenatal care	171	1.5
Excessive ear wax	11	0.8	Immunisation/vaccination	150	1.3
Fever	11	0.8	Vomiting	147	1.3
Subtotal	1102	76.2	Subtotal	8284	67.1
Total	1446	100.0	Total	11,562	100.0

Note: to rule out tuberculosis = suspected tuberculosis

Table 4 describes the management provided during these encounters. Most encounters resulted in prescription of medications, where 76.5 % of 10,830 encounters received at least one medication during visit. In total, there were 24,196 medications prescribed with an average rate of 223 medications per 100 encounters. The prescription rate was higher in private clinics (229 per 100 encounters) compared to public clinics (170 per 100 encounters). Medications for chronic diseases, namely hypertension, diabetes mellitus and lipid disorder were prescribed for a duration range of 14 to 180 days in public clinics and between 14 and 56 days in private clinics. Investigations and procedural rates were about two times higher in public clinics. Similarly, encounters scheduled for subsequent follow-up visit was significantly higher in public clinics (72.1 %) compared to private clinics (22.5 %). The referral rates however, were relatively low in both sectors.

**Table 4:** Management provided in primary care clinics

	Overall		Public clinics		Private clinics		
	(*n* = 10,830)		(*n* = 1067)		(*n* = 9763)		
	No. of encounter	Percentage	No. of encounter	Percentage	No. of encounter	Percentage	*P-*value*
Medication							
Prescribed	8279	76.5	805	75.4	7474	76.6	0.8919
Investigation	2976	27.5	587	55.0	2389	24.5	0.0007
Procedure	2199	20.3	464	43.5	1735	17.8	<0.0001
Visit disposition							
Referral	438	4.0	99	9.3	339	3.5	0.0174
Follow-up	2963	27.4	769	72.1	2194	22.5	<0.0001
Medical leave certificate issued	2042	18.9	91	11.4	1951	22.0	0.0100

*Chi-square test for differences between public and private clinics, P-value < 0.05 considered as significant

## Discussion

Foreigners accounted for 7.7 % of all primary care consultations and most encounters were from private clinics. This was expected as some foreign workers have access to private clinics through company panel clinics or some may find easy access to private clinics with shorter waiting time and longer operating hours which run beyond office hours and on weekends [36] as was found among migrant workers in Thailand [37]. There were differences in the type of illnesses encountered in public and private clinics; commonest conditions sought in public clinics among foreigners were obstetric cases (37.7 %) and chronic diseases (15.5 %) whereas health problems encountered in private clinics were mostly minor conditions or acute illnesses. This is similar to findings previously observed in studies of general patient population in Malaysia where public clinics in general handled more chronic diseases while private clinics saw more cases of acute and minor ailments [38, 39].

We found that obstetric cases were frequently encountered, particularly in public clinics where nearly half of the encounters are related to pregnancy. The preferences for government healthcare facilities over private sector for obstetric problems is similar to the findings from the study of migrant workers in Thailand [37]. The preponderance of pregnancy-related cases in public clinics might be related to cost, as private healthcare charges are fee-for-service. Although most foreigners take up insurance coverage on entering the country, most often it does not cover care related to pregnancy. This finding highlights the prevalence of obstetric cases and suggest the need for maternal health services among foreigners. This is in line with a previous study conducted in Sabah – a state in Malaysia with the highest number of foreigners’ population, which reported that the use of antenatal care services among foreigners during pregnancy was fairly high (91.0 %), although the proportion of those who did not obtain antenatal care was also significantly higher (8.1 %) when compared to Malaysian citizens (2.9 %) [40]. Studies also showed that foreign migrants tend to initiate care at a later stage of pregnancy and this could be attributed to unfamiliarity with the health system or inadequate access to healthcare facilities [9, 40–42]. All pregnant women should have access to adequate antenatal care as appropriate management could reduce maternal mortality rates and birth complications [43]. It is also interesting to know which category of foreigners these pregnant women fall into considering that pregnancy is one of the conditions that precludes employment of a foreigner in the non-professional category of work and these blue collar workers are not allowed to bring their family members to live in this country during the term of employment [22]. Our study however, did not collect details on their socioeconomic or occupational status hence this could not be determined.

Beside obstetric cases, our study found that chronic non-communicable diseases, namely hypertension, diabetes mellitus and lipid disorder were frequently encountered in public clinics as they amounted to 15.5 % of the diagnoses. While these conditions were also managed in private clinics, the percentage was smaller at 4.5 % because private clinics had more cases of acute minor ailments. A study by Leong in 2006 which reviewed eight years of patient records in a private clinic in Malaysia revealed that hypertension comprised 34.1 % of medical problems and was the commonest medical problem detected during pre-employment medical examination of foreign workers [44]. For foreign workers, they are expected to be healthy for employment, although the rules allow foreign workers with chronic illnesses to be employed if they are not debilitating. Those with diseases need to be treated and there is a growing concern with regards to management of chronic diseases cases among foreigners in public clinics and how it might impose demand on the resources of the public healthcare system which is already strained. Unlike acute illnesses, treatment of chronic diseases is long term and incurs higher cost, which partly explains the preferences for public sector [45]. In this study, we found that the maximum duration of medications for chronic diseases supplied by public clinics was for six months. However, there is a new directive from Ministry of Health Malaysia to limit the supply of medications for chronic non-communicable diseases to foreigners for only five days for each consultation; the rest will have to be sourced at the patients’ own cost from the private sector [46]. This move is seen as part of an effort to reduce public healthcare spending and limiting the government health subsidies to foreigners, in light of prevailing government view point that foreigners took up 30 to 40 % of the healthcare subsidies by Malaysian government [47]. Our study however, was conducted before this policy was implemented thus future study to look into the effect of the changes in policy and further cost analysis is necessary.

A substantial proportion of encounters (13.5 %) from the private clinics are for general medical examinations. The relative frequency for general medical examination, particularly in private clinics, was to be expected because health assessment is mandatory for foreigners who are planning to work, study or seek residency in the country [22, 48]. Furthermore, most of these encounters were for pre-employment or annual health examinations required for renewal of their work permits. This requirement is conducted in private clinics registered with the Foreign Workers’ Medical Examination Monitoring Agency (FOMEMA), the agency responsible for coordinating and monitoring the task [22, 49]. The workers are screened for any condition that renders them medically unfit for employment, as mandated by the Ministry of Health Malaysia [50].

We noted six encounters with a diagnosis of tuberculosis and 45 with suspected tuberculosis. Although they formed less than 1.0 % of the encounters, the actual prevalence may be higher because more than 60.0 % of tuberculosis cases are diagnosed and treated in public hospitals [51] and therefore not included in this study. A review of Malaysian Tuberculosis Registry showed that 14.2 % of the 21,582 tuberculosis patients were foreigners [52]. Studies in several countries have shown that tuberculosis is a common infectious disease diagnosis reported among foreign migrants [37, 53, 54]. Transmission of tuberculosis is often linked to migration of people from high risk countries [55]. Hence foreign workers are screened for infectious diseases before they enter the country, although there are also many who bypass the system which further complicates the control programme. The responsibility therefore rests on physicians, especially the primary care providers as the first point of care, to have a high index of suspicion for undetected tuberculosis cases and subsequent treatment and monitoring.

Acute and minor illnesses reported among foreigners in our study population were mainly on febrile illness and respiratory symptoms, injuries, musculoskeletal complaints, and gastrointestinal problems. The frequent presentation of these conditions were also reported in studies from other countries that sampled foreign migrant workers [12, 18, 37]. A previous local study on illness pattern of foreign workers conducted in 2002 indicated that the most frequent medical complaints were injuries or accidents (19.6 %), musculoskeletal (18.0 %), gastrointestinal (16.7 %) and fever (14.5 %) [11]. By contrast, our study identified higher rates of respiratory ailments while injuries or accidents appeared to be less frequent. This could be explained by the fact that our study described cases managed in primary care clinics only, while the previous report included cases managed in the emergency departments of hospitals. Moreover, our study included all foreigners regardless of employment status or age; the health problems encountered would therefore be more diverse.

### Limitations

Research on foreigners is challenging considering the sparse epidemiologic data available for this population in Malaysia. Hence the NMCS study provides a source and opportunity to describe the clinical profiles of a large sample of foreigners. However, our study also has several limitations. Firstly, the health problems were based on the cases managed during the encounters; patients may have other existing diseases which were not highlighted during the encounters. Secondly, most of the encounters were from private clinics while those in public clinics accounted for only 9.8 % of the study population. The findings should be interpreted according to sector as overall results combining both sectors will skew the results towards private sector. Thirdly, the study did not distinguish whether the foreigners were workers, students or tourists. Finally, this study included data from three states and two regions in Malaysia and the data ought to be interpreted with caution as it might not represent the true foreigners’ population in this country.

## Conclusion

More foreigners were seeking primary medical care from private clinics. The encounters were for general medical examinations and acute minor ailments. Those who sought care from public clinics were for obstetric problems and chronic diseases. Medications were prescribed to two-thirds of the encounters in both sectors while other interventions, namely laboratory investigations, medical procedures and follow-up appointment had lower rates in private clinics. Encounters in private clinics for general medical examinations and acute illnesses were expected as the foreigners are generally of young working group and are expected to have mandatory medical checks. However, the preponderance of obstetrics seen in public clinics suggests a need for improved access to maternal care and pregnancy related care. This has implication on policy and health care provision and access for foreigners and future studies are needed to look into strategies to solve these problems. The Ministry of Health Malaysia has recently introduced policy to impose gradual increment of medical fees for foreigners in public healthcare facilities [56]. Further research is needed to explore if this cost imposition on foreigners seeking healthcare in the public sector would create barriers to accessing healthcare or delay presentation to services to the detriment of health outcomes.

## Abbreviations

ATC, Anatomical Therapeutic Chemical; ICPC, International Classification of Primary Care; IQR, interquartile range; NMCS, National Medical Care Survey; RFE, reason for encounter; SD, standard deviation; URTI, upper respiratory tract infection

## Appendix

Additional table contained information on characteristics of respondents and non-respondents of the private clinics.

**Table 5:** Comparison of gender, age and years of practice between the respondents and non-respondents for the private clinics

	Respondents	Non-respondents	Test statistics	*P*-value
Gender, n (%)				
Male	80 (54.8 %)	144 (63.7 %)	2.9475^a^	0.086
Female	66 (45.2 %)	82 (36.3 %)		
Age, mean (SD)	51.1 (11.5)	51.4 (11.1)	0.1987^b^	0.8426
Years of practice, mean (SD)	21.9 (12.5)	22.5 (10.1)	0.4482^b^	0.6544

*SD* standard deviation

^a^Chi-square test, P-value <0.05 considered as significant

^b^t-test, P-value <0.05 considered as significant
